# Asymptomatic Severe Acute Respiratory Syndrome Coronavirus 2 (SARS-CoV-2) Infection in a Rehabilitation Facility: Evolution of the Presence of Nasopharyngeal SARS-CoV-2 and Serological Antibody Responses

**DOI:** 10.1093/infdis/jiaa610

**Published:** 2020-10-16

**Authors:** Benjamin H L Harris, Mohamed Zuhair, Matteo Di Giovannantonio, Carolina Rosadas, Maryam Khan, Charlotte-Eve Short, Thilipan Thaventhiran, Rachael Quinlan, Andrew Taylor, Ronan Calvez, Graham P Taylor, Richard S Tedder, Myra O McClure, Michael Fertleman

**Affiliations:** 1 The Wellington Hospital, Circus Road, St John’s Wood, London, United Kingdom; 2 Department of Infectious Disease, Faculty of Medicine, Imperial College London, London, United Kingdom; 3 Computational Biology and Integrative Genomics, Department of Oncology, University of Oxford, Oxford, United Kingdom; 4 Micropathology, University of Warwick Science Park, Coventry, United Kingdom; 5 Cutrale Perioperative and Ageing Group, Department of Bioengineering, Imperial College London, London, United Kingdom

**Keywords:** COVID-19, SARS-CoV-2, asymptomatic, anti-RBD, SARS-CoV-2 receptor binding domain (RBD), antibodies to nucleoprotein, anti-NP, Imperial Hybrid DABA

## Abstract

At the start of the UK coronavirus disease 2019 epidemic, this rare point prevalence study revealed that one-third of patients (15 of 45) in a London inpatient rehabilitation unit were found to be infected with severe acute respiratory syndrome coronavirus 2 (SARS-CoV-2) but asymptomatic. We report on 8 patients in detail, including their clinical stability, the evolution of their nasopharyngeal viral reverse-transcription polymerase chain reaction (RT-PCR) burden, and their antibody levels over time, revealing the infection dynamics by RT-PCR and serology during the acute phase. Notably, a novel serological test for antibodies against the receptor binding domain of SARS-CoV-2 showed that 100% of our asymptomatic cohort remained seropositive 3—6 weeks after diagnosis.

The disease caused by severe acute respiratory syndrome coronavirus 2 (SARS-CoV-2), coronavirus disease 2019 (COVID-19) is complex, displaying variable clinical symptoms and disease severity. On this spectrum, most patients develop mild symptoms. Predominant features of the disease include a dry cough, fever, sore throat, and loss of taste and smell [1]. In some cases, serious complications develop, including septic shock, severe pneumonia, renal failure, and atypical acute respiratory distress syndrome [2]. Age, sex, ethnicity, and comorbid conditions (including obesity and cardiovascular, cerebrovascular, endocrine, digestive, and respiratory disease) have all been associated with poorer prognosis [1].

Asymptomatic carriers are well documented and have been implicated in disease transmission. On the Diamond Princess cruise ship, SARS-CoV2 infection was diagnosed in 634 of the 3711 passengers [3], of whom 17.9% were asymptomatic. In other examples, the proportion of those asymptomatically infected ranges between 40% and 45% [4].

Current testing strategies revolve around the use of reverse-transcription (RT) polymerase chain reaction (PCR) tests from nasopharyngeal swab samples. However, in the absence of viral cultures, RT-PCR results can be difficult to relate to infectiousness [5]. Furthermore, the ability to detect past infection or host immunity by this route is not well defined [6]. If undertaken within the correct time frame, serological testing can potentially detect both active and past SARS-CoV2 infection [7]. Consequently, the use of serological data has controversially been mooted as “a way out of lockdown” by offering the possibility of “immunity passports” [8]. Trials are also underway to establish whether the plasma from convalescent individuals with high levels of neutralizing antibody may help treat unwell patients [9].

SARS-CoV2 was initially described in Wuhan, China, in December 2019 [10]. At the beginning of March 2020, the number of confirmed cases of SARS-CoV2 in London rose from 25 to >11 000 [11]. During the third week in March, managers of a rehabilitation facility in London arranged for all inpatients to be tested for the presence of SARS-CoV2. Most hospitals in the United Kingdom at this stage were testing only symptomatic patients, making this study unique. This approach permitted description of the clinical and immune responses in a group of asymptomatic patients and enabled a rough estimate of the burden of asymptomatic carriers within London hospitals at that time. In addition, we present prospective hematological, biochemical, and serological measures taken over a 4-week period (weeks 3–6 after infection was diagnosed) in a group of these asymptomatic SARS-CoV2–infected individuals.

## METHODS

After ethical approval from the rehabilitation hospital, all samples were donated with informed written consent to the local University Communicable Disease Research Tissue Bank (NRES SC/20/0226). The use of the samples for research was approved by the Tissue Bank Steering Committee in accordance with National Research Ethics Service procedures.

### Patient Cohort

All participants were inpatients in a specialist adult neurological rehabilitation hospital in northwest London. In this facility, each patient is managed in his or her own room. Between 21 March and 31 March 2020, trained nursing staff obtained nasopharyngeal swab samples from all 45 patients. Patients found to be positive for SARS-CoV-2 were transferred to an isolation ward. As part of routine management, these patients were closely monitored for signs and symptoms of COVID-19 and had weekly blood tests. Additional swab samples were taken at least weekly, beginning the third week after diagnosis. Patients remained in the isolation ward until they had 2 consecutive RT-PCR–negative swab samples. Serum samples for anti–SARS-CoV2 antibody were first obtained 3 weeks (18–24 days) after the first positive RT-PCR result and then obtained weekly for 4 weeks (at 3, 4, 5, and 6 weeks after diagnosis).

### RT-PCR Protocol

To identify SARS-Cov2 infection, RNA from all nasopharyngeal swab samples was extracted within 24 hours. Diagnostic testing for SARS-CoV-2 was performed by Micropathology (**University of Warwick Science Park**). Nucleic acid was extracted using the Maxwell HT 96 NA extraction kit (Promega) and the KingFisher FLEX platform (ThermoFisher Scientific). RT-PCR primers testing were as recommended by the US Centers for Disease Control and Prevention [12]. RT-PCR was performed using a Roche LightCycler 480 instrument on the following cycle: RT (50°C for10 minutes) and polymerase activation (95°C for 2 minutes), followed by PCR (45 cycles of 95°C for 5 seconds and 55°C for 20 seconds). Samples with a cycle threshold (Ct) value >40 and no history of positive detection were retested. DNA from baculovirus *Adoxophyes orana* granulovirus was used as an internal control. Any samples failing to amplify (or showing late or weak amplification) underwent re-extraction, and PCR was repeated. Patients with 2 consecutive negative RT-PCR results were defined as SARS-CoV2 negative.

### Detection of Anti–SARS-CoV-2 Antibodies

Serological screening for SARS-CoV2 antibody was carried out at Imperial College London. Antibody to the SARS-CoV2 receptor binding domain (RBD) was detected using a 2-step sequential “in-house” double antigen-binding assay, using S1 antigen on the solid phase and enzyme-labeled RBD in the fluid phase. The specificity was 100% (95% confidence interval, 99.6%–100%) (defined by testing 825 serum samples that predated the epidemic); the sensitivity, 98.91% (96.8%–99.8%) (using 276 serum samples from RT-PCR–confirmed individuals) [13]. This assay detects total immunoglobulins for SARS-CoV2, avoiding problems associated with pure IgM or IgG assays, particularly where the elucidated time to seroconversion from IgM to IgG in SARS-CoV2 infection is still under debate in different patient groups [14]. The assay cutoff was calculated from receiver operating characteristic curve analysis, and serum reactivity normalized by using the signal-to-cutoff ratio (S/CO), the ratio of optical density values generated in a sample to the cutoff optical density value. A sample was considered antibody positive if the S/CO was >1.

### Statistical Analysis

Analyses were carried out using Python software, version 3.7. Two-sided Mann-Whitney-Wilcoxon tests (paired and unpaired) were used as appropriate. Bonferroni correction was used to correct for the possibility of type 1 error. Spearman rank correlation coefficient was used to correlate Ct values and anti-RBD.

## RESULTS

SARS-CoV2 RNA was detected in 15 of 45 patients. Twelve consented to participate; of these, 4 were discharged during the study period and were lost to follow-up. Data are presented on the 8 patients who were followed up for 6 weeks, including 5 male and 3 female patients, with a mean age of 51 years. Regarding comorbid conditions, 4 of the 8 had had a stroke, and 3 had undergone neurosurgery. Of 2 patients with type II diabetes, 1 also had hypertension, chronic obstructive pulmonary disease, and chronic kidney disease. All reported either no symptoms or, on questioning, very mild symptoms not requiring medical intervention. Five reported a mild fever, but on repeated tympanic measurements over the study period, no patient had a temperature ≥38°C. Two reported a mild cough lasting <24 hours.

Biochemical and hematological markers were evaluated at baseline and throughout the 2-month period. The only abnormalities detected were mildly reduced lymphocyte counts in 2 patients, which returned to normal by the third week after diagnosis. Biochemistry showed mildly deranged liver function results in 1 patient, attributed to a concurrent course of antibiotics for an unrelated bacterial infection.

### Detection of SARS-CoV-2 RNA

**Figure 1.**
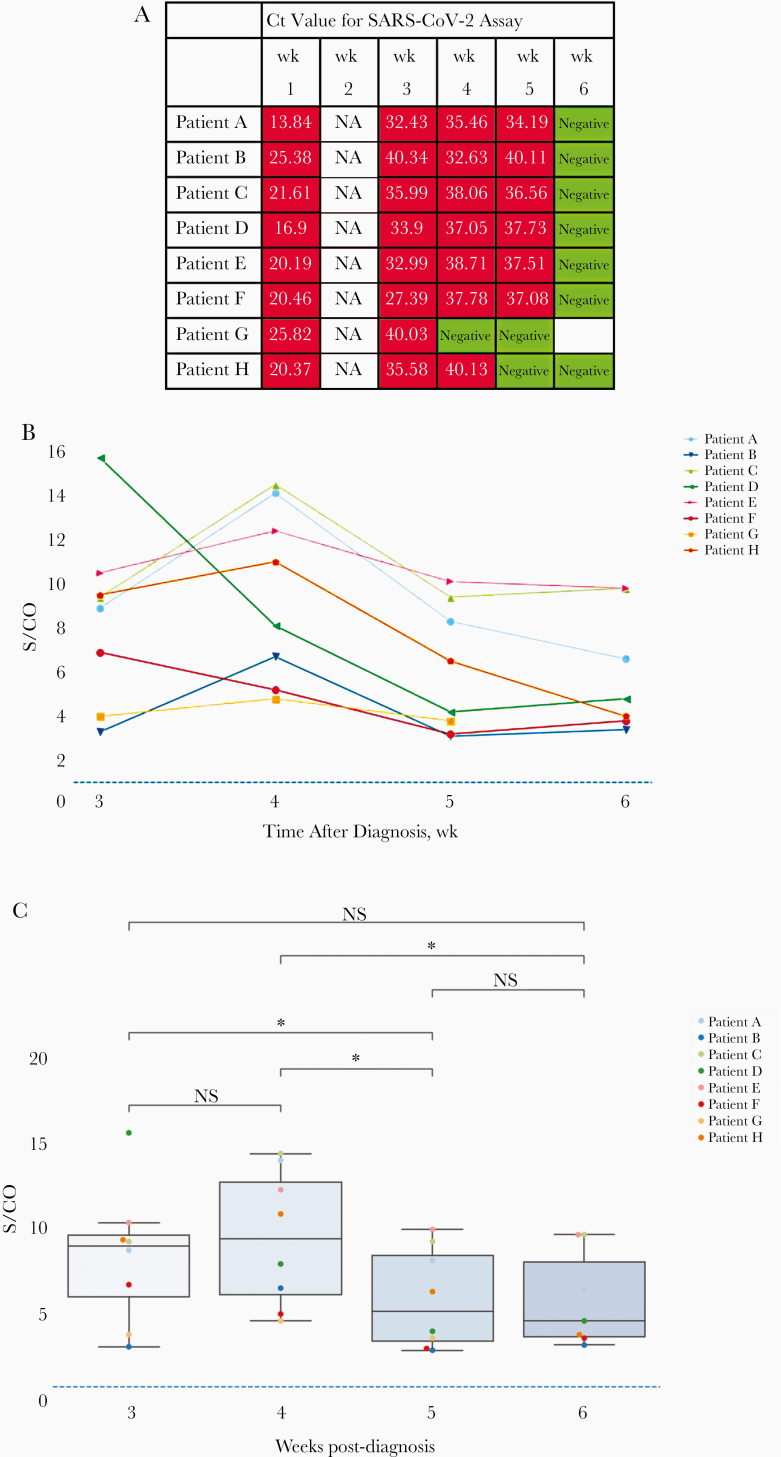
*A,* Polymerase chain reaction (PCR) cycle threshold (Ct) results for severe acute respiratory syndrome coronavirus 2 (SARS CoV-2) over the study period, from nasopharyngeal swab samples. Red indicates positive and green negative for SARS-CoV2. *B,* Serum antibody to the SARS-CoV2 receptor binding domain (anti-RBD) over a 4-week period (weeks 3–6 after PCR diagnosis). *C,* Box plots showing individual serum anti-RBD results. Abbreviations: NA, not available; NS, not significant; S/CO, signal-to-cutoff ratio (ratio of optical density in a sample to the cutoff optical density). *.01 < *P* ≤ .05 (2-sided Mann-Whitney-Wilcoxon test).

In this point prevalence study, the median Ct at which signal amplification is detected above the Ct at initial sampling was 20.4 (interquartile range [IQR], 19.4–22.5). The Ct increased significantly at the second testing point (2 weeks later), to a median of 34.7 (IQR, 32.8–37) (*P* < .001), representing about a 4-log_10_ reduction in SARS-CoV2 viral load in in the nasopharyngeal swab samples. There was a further log_10_ reduction over the next 7 days (median Ct, 37.8; IQR, 36.2–38.4), with SARS-CoV2 RNA still detected in 7 of 8 patients. Thereafter SARS-CoV2 was not detected. Participants remained swab sample positive for a mean of 32 days (range, 15–46 days) before 2 sequential negative swab samples. Individual results are presented in Figure 1A.

### Antibody Response

Antibodies to RBD were present in all 8 patients at the first serum sample (3 weeks after SARS-CoV2 RNA was first detected). The weekly antibody response is presented in Figure 1B. In 6 of 8 patients, the highest measured S/CO was during week 4. Anti-RBD was still detected in all 8 patients during week 6, with no significant change in the S/CO.

Figure 1C displays individual antibody levels for the 8 patients, together with a statistical comparison. There were statistically significant drops in total immunoglobulin levels between weeks 3 and 5 (*P* = .01), weeks 4 and 5 (*P* = .01) and weeks 4 and 6 (*P* = .049) after diagnosis. The changes between weeks 3 and 4 and between weeks 4 and 5 survived stringent Bonferroni correction. Spearman rank correlation between Ct values at week 1 and anti-RBD serum levels at week 3 yielded a negative correlation very close to statistical significance (r = -0.69; *P* = .06). Other correlations between Ct values in subsequent weeks were in a negative direction but were further from statistical significance.

## DISCUSSION

The evolution of serological and biochemical changes is currently not well defined in asymptomatic patients with SARS-CoV-2 infection. In March 2020, at the beginning of the COVID-19 outbreak in London, testing all patients in 1 institution for SARS-CoV2 RNA was unique; guidance suggested testing only symptomatic patients. Therefore, the question remained for healthcare planners whether there was infectious virus shedding among asymptomatic patients. We contribute a rare point prevalence study of asymptomatic patients with longitudinal antibody and RT-PCR follow-up. This work shows that 15 of 45 rehabilitation patients (33%) in 1 hospital were infected when the number of symptomatic COVID-19–positive patients in London was 11 000 (about 0.01% of the London population) [11]. This implies that the number who had contracted the virus was higher in the immediate postlockdown period than data from early in the UK pandemic suggested.

The only comparable published study is on an asymptomatic cohort of 37 patients from Wuhan, China [15]. Using a chemiluminescence enzyme immunoassay for IgG, these workers found that 40.0% (12 of 30 asymptomatic individuals) became seronegative at 8 weeks after PCR diagnosis [15]. Our study provides the first longitudinal estimates of serum total immunoglobulin levels from weeks 3–6 after diagnosis. In all 8 asymptomatic patients, an antibody response developed within 3 weeks of first PCR-positive result, and this persisted throughout the 4-week follow-up period. Although antibody levels fluctuated during the study period, 100% of our cohort remained seropositive for anti-RBD between 3 and 6 weeks after PCR diagnosis. This work suggests that 3–6 weeks after PCR positivity is a suitable time frame to check for a serological response in asymptomatic patients.

## Notes

***Financial support.*** This work was supported by the Department of Infectious Disease, Imperial College London.

***Potential conflicts of interest.*** C. R., R. S. T., and M. O. M. declare an interest in the Imperial Hybrid double antigen-binding assay (patent file IRN.FID4816059). All other authors report no potential conflicts. All authors have submitted the ICMJE Form for Disclosure of Potential Conflicts of Interest. Conflicts that the editors consider relevant to the content of the manuscript have been disclosed.
